# Astragaloside IV attenuates hypoxia-induced pulmonary vascular remodeling via the Notch signaling pathway

**DOI:** 10.3892/mmr.2021.12547

**Published:** 2021-11-29

**Authors:** Jiamei Yao, Xia Fang, Cui Zhang, Yushu Yang, Dongsheng Wang, Qiong Chen, Guangwei Zhong

Mol Med Rep 23: Article no. 89, 2021; DOI: 10.3892/mmr.2020.11726

Subsequently to the publication of the above article, an interested reader drew to the authors’ attention that, in Fig. 4A on p. 6, the ‘T’ and ‘DAPT’ data panels appeared to contain some of the same data, although one of the panels appeared to have been rotated through 180° relative to the other.

The authors have re-examined their original data, and have realized that this figure was assembled incorrectly. Subsequently, the authors have reperformed the experiments that were concerned with the immunohistochemical detection of PCNA in rat lung tissue samples, and the new results for Fig. 4A are shown in a corrected version of Fig. 4 on the next page. Note that the errors made in assembling the original version of this figure did not quantitatively affect either the results or the overall conclusions reported in this paper. The authors are grateful to the Editor of *Molecular Medicine Reports* for allowing them this opportunity to publish a Corrigendum; furthermore, they apologize to the readership for any inconvenience caused.

## Figures and Tables

**Figure 4.**
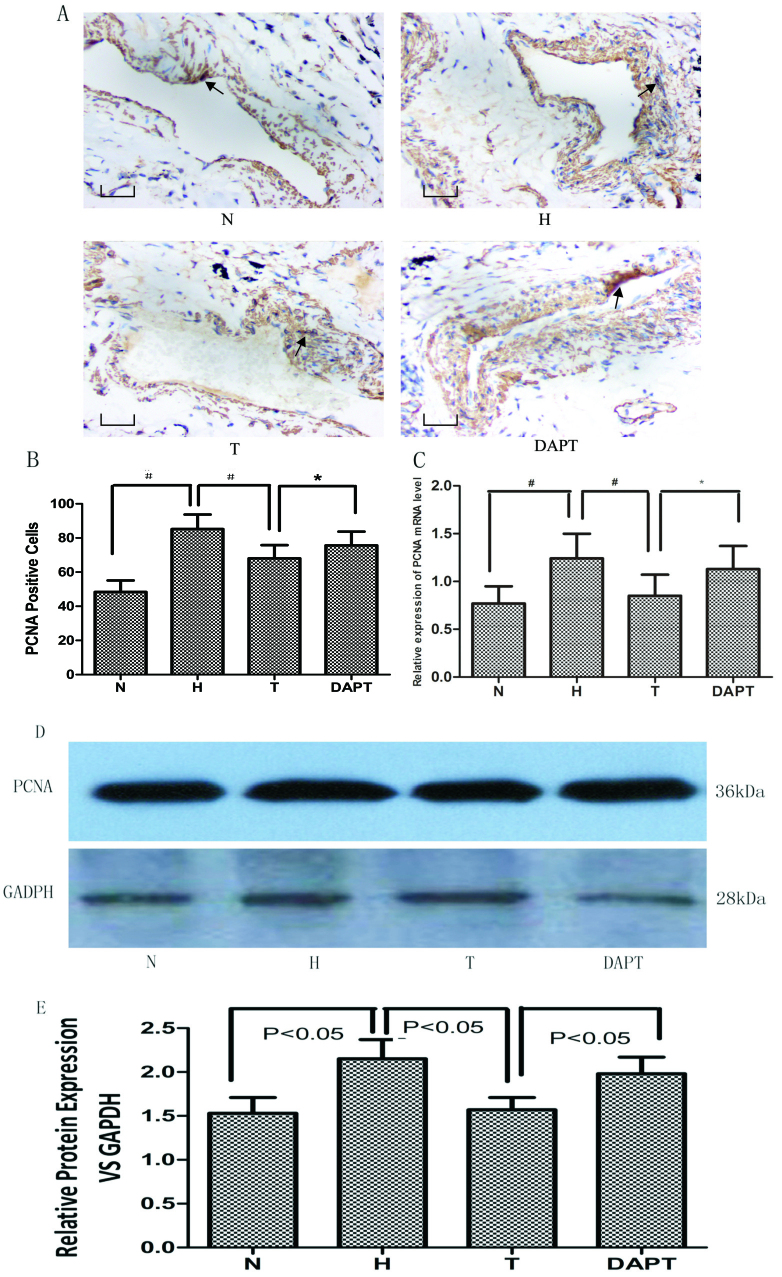
Effect of Astragaloside IV on PCNA expression. (A) PCNA expression was primarily detected in the pulmonary arteries (The black arrow refers to the positive expression of PCNA protein in pulmonary vascular tissue). Scale bar, 10 µm. Magnification, ×100. (B) Quantification of PCNA+ cells in lung sections. (C) PCNA mRNA expression in lung sections. PCNA protein expression levels in pulmonary artery smooth muscle cells were (D) determined via western blotting and (E) semi-quantified. *P<0.05; ^#^P<0.01. PCNA, proliferating cell nuclear antigen; N, normoxia; H, hypoxia; T, treatment; DAPT, N-[N-(3,5-difluorophenacetyl)-L-alanyl]-S-phenylglycinet-butyl ester.

